# Evaluation of a strawberry fermented beverage with potential health benefits

**DOI:** 10.7717/peerj.11974

**Published:** 2021-08-23

**Authors:** Zhiqiao Zhao, Xulong Wu, Hong Chen, Yuntao Liu, Yirong Xiao, Hui Chen, Zizhong Tang, Qingfeng Li, Huipeng Yao

**Affiliations:** 1 College of Life Sciences, Sichuan Agricultural University, Ya’an, China; 2 Chengdu Agricultural College, Chengdu, China; 3 College of Food Sciences, Sichuan Agricultural University, Ya’an, China; 4 Sichuan Agricultural University Hospital, Ya’an, China

**Keywords:** Fermentation, Fruit fermented beverage, Antioxidant Capacity, Pathogenic bacteria, Anti-biofilm formation, Sensory analysis, Strawberry, Probiotics, Fourier transform infrared (FTIR) analysis, Fluorescence microscopy

## Abstract

**Background:**

Functional fermented beverages are popular worldwide due to their potential to promote health. Starter culture is the main determinant of the final quality and flavor of fermented beverages. The co-cultivation of lactic acid bacteria (LAB) and yeast makes a significant contribution to the safe flavor of fermented beverages. However, the research on the potential of antioxidant, antimicrobial, and anti-biofilm formation of strawberry fermented beverage obtained by combining the LAB and yeast as starter cultures has not been well explored.

**Methods:**

In this study, LAB and yeast were combined as starter culture to obtain strawberry fermented beverage. Fourier transform infrared (FTIR ) spectroscopy was used for the qualitative analysis of the fresh strawberry juice and fermented beverage. From the changes in antioxidant content, free radical scavenging ability, total superoxide dismutase (T-SOD) activity and total antioxidant capacity (T-AOC) to evaluate the antioxidant capacity of fermented beverage *in vitro*. The antibacterial ability was tested by the Oxford cup method. The biofilms of *Escherichia coli* ATCC 25922, *Staphylococcus aureus* ATCC 6538 under fermented beverages treatment was observed by Fluorescence microscope. In addition, sensory analysis was conducted in this study.

**Results:**

In this study, the absorption peaks of Fourier transform infrared between 1,542 cm^−1^ and 976 cm^−1^, suggest the existence of organic acids, sugars and ethanol. The total phenols and total flavonoids content decreased by 91.1% and 97.5%, respectively. T-SOD activity increased by 33.33%.The scavenging ability of fermented beverage on superoxide anion free radicals was enhanced, and the scavenging ability on DPPH free radicals, hydroxyl free radicals, and ABTS free radicals was weakened. However, the T-AOC increased from 4.15 ± 0.81 to 8.43 ± 0.27 U/mL. Fermented beverage shows antibacterial activity against four pathogens. The minimum inhibitory concentration (MIC) values of *Escherichia coli* ATCC 25922 and *Staphylococcus aureus* ATCC 6538 were 0.05 mL/mL and 0.025 mL/mL, respectively, and the minimum bactericidal concentration (MBC) were both 0.2 mL/mL. It was observed by fluorescence microscope that the green fluorescence area of the two biofilms is greatly reduced after being treated with fermented beverage. Sensory analysis results show that the average scores of fermented beverage in color, appearance and taste were increased. The overall impression and flavor were decreased.

**Conclusion:**

These results demonstrated that strawberry fermented beverage has potential benefits such as an antioxidant, antibacterial, and anti-biofilm formation, providing the potential for the fermented beverage to become promising candidates for natural antioxidants, antibacterial agents and anti-biofilm agents.

## Introduction

Strawberry (*Fragaria x ananassa Duch*) is one of the most popular berries around the world and is rich in a variety of nutrients such as minerals, vitamin C, vitamin E, phenolics, flavonoids, β-carotene, folate and potassium (Zhang et al., 2012; Peretto et al., 2014; Skrovankova et al., 2015). However, strawberry is an extremely perishable fruit because of its high respiration rate and high level of water content, being vulnerable to mechanical damage, water loss, and microbial infections during storage (Sanz et al., 1999; Perkins-Veazie, 2010). Selection of ideal processing technology to prolong the shelf life of strawberries and increase the added value are important problems to be solved. With the rise of vegetarianism and the discovery of the negative effects of probiotic dairy products, such as lactose intolerance, an increase in cholesterol content, and a milk protein allergy (Camargo Prado et al., 2015), consumers are focusing on non-dairy probiotic products that are high in nutrition and free of cholesterol and lactose (Pereira et al., 2017). In the development of non-dairy probiotics, the development of functional fermented beverages, use fruit and vegetable juice as substrate, has attracted people’s interest (Pereira, Maciel & Rodrigues, 2011), because fruit and vegetable juice is a good source of nutritional compounds (such as carbohydrates, dietary fibers, vitamins, minerals), can be used as a carrier of probiotics (Septembre-malaterre, Remize & Poucheret, 2018). This has been proven with cucumber juice (Tamang & Tamang, 2010) pomegranates juice (Mantzourani et al., 2019), orange juice (Escudero-López et al., 2015), pineapple juice (Chanprasartsuk et al., 2010) as well as others (Dias et al., 2018), and this type of fermented beverages tastes appeal to people of all ages as they are considered healthy and refreshing (Pereira, Maciel & Rodrigues, 2011). Fermentation is a biotransformation process that is affected by two main factors: starter cultures or native microorganisms and substrates, and other inherent factors such as temperature and pH conditions (Yunita & Dodd, 2018). Therefore, the appropriate starter cultures and substrate should be selected before the preparation of fermented beverage. Considering the nutritional value and sensory characteristics of the fruit and vegetable fermented beverage, LAB and yeast were combined as starter cultures because effect of multistrain or multispecies probiotic beverages may provide greater beneficial effects than monostrain culture (Marsh et al., 2014). When yeast and LAB are co-cultured, it can not only promote the growth of either group of microbes but also the fermented product has flavor and good organoleptic properties (Mukisa et al., 2017). In this context, strawberry juice rich in a variety of nutrients molecules can be considered fermented into functional fermented beverages to extend the shelf life.

During the process of cell metabolism, excessive production of free radicals can cause a series of diseases, such as aging, obesity, arteriosclerosis, etc. (Hassan, Ghareb & Azhari, 2017). Therefore, the balance and stability of free radicals is very important for maintaining the body. At present, the extensive use of antibiotics poses a great threat to environmental safety (Gao et al., 2012), and the resistance of bacteria to antibiotics has become very obvious (Jankauskaite et al., 2016). Some of the common multidrug-resistant microorganisms are *Escherichia coli*, *Staphylococcus aureus* and *Pseudomonas aeruginosa*, *Bacillus subtilis* (Alekshun & Levy, 2007). *Staphylococcus aureus* is a Gram-positive bacterium with a variety of virulence factors that can cause some symptoms such as pulmonary, osteoarticular and skin and soft tissue infections, as well as *Bacillus subtilis*, which can cause food spoilage (Tong et al., 2015; Wu et al., 2020). *Escherichia coli* and *Pseudomonas aeruginosa* are Gram-negative microorganisms, related to diseases in humans and animals, such as gastroenteritis, urinary infections, peritonitis and meningitis (Picoli et al., 2017). Biofilm is one of the main reasons for the development of multidrug resistance in most bacteria. In addition, the appearance of biofilm on kinds of medical equipment and implants such as contact lenses, heart valves, artificial joints and among others usually causing both systemic and local infections (Vishwakarma & Vavilala, 2019). The selection and development of novel antioxidants, antibacterial agents or anti-biofilm agents is of great significance in this context. Fruit and vegetable functional fermented beverages can be used as a new nature candidate because of their potential health benefits. Hashemi et al. (2017) showed that fermentation improves the chemical properties such as antioxidant activity as well as antibacterial properties compared with unfermented sweet lemon juice, and the fermented juice could be used as a nondairy functional food product. This was consistent with the results obtained after fermentation of immature pear fruits (Lee et al., 2016). Ayed, Ben Abid & Hamdi (2017) also observed the same result, that is, the antioxidant capacity and antibacterial capacity against the tested microorganism of red grape juice after fermentation with kombucha consortium were enhanced. In addition, other fermented beverages such as pomegranates (Mantzourani et al., 2019), cupuassu pulps (Pereira et al., 2017), red beetroot (Vanajakshi et al., 2015), and other fruits as fermentation carriers also have potential health benefits such as anti-oxidant, antibacterial and other health effects. However, to the best of our knowledge, no studies have considered the use of fermented fruit and vegetable beverages as anti-biofilm formation inhibitor, and research on fermented strawberry beverages has focused on the physiochemical properties (Ordóñez et al., 2015; Hornedo-Ortega et al., 2016; Park et al., 2017; Yang et al., 2021), and the research on potential health benefits of strawberry fermented beverage is relatively poor.

Therefore, the current study aims to evaluate the potential health effects (antioxidant, antibacterial and anti-biofilm potential) of strawberry fermented beverages. In addition, its sensory analysis has also been studied. Our study will help pave the way to develop novel natural antioxidants, antibacterial agents, and anti-biofilm properties.

## Materials & Methods

### Schematic overview of the experimental program

This study mainly used the non-spontaneous fermentation method to obtain strawberry fermented beverage, and then explored the potential health promotion benefits of strawberry fermented beverage. The specific scheme is as follows.

**Figure 1:**
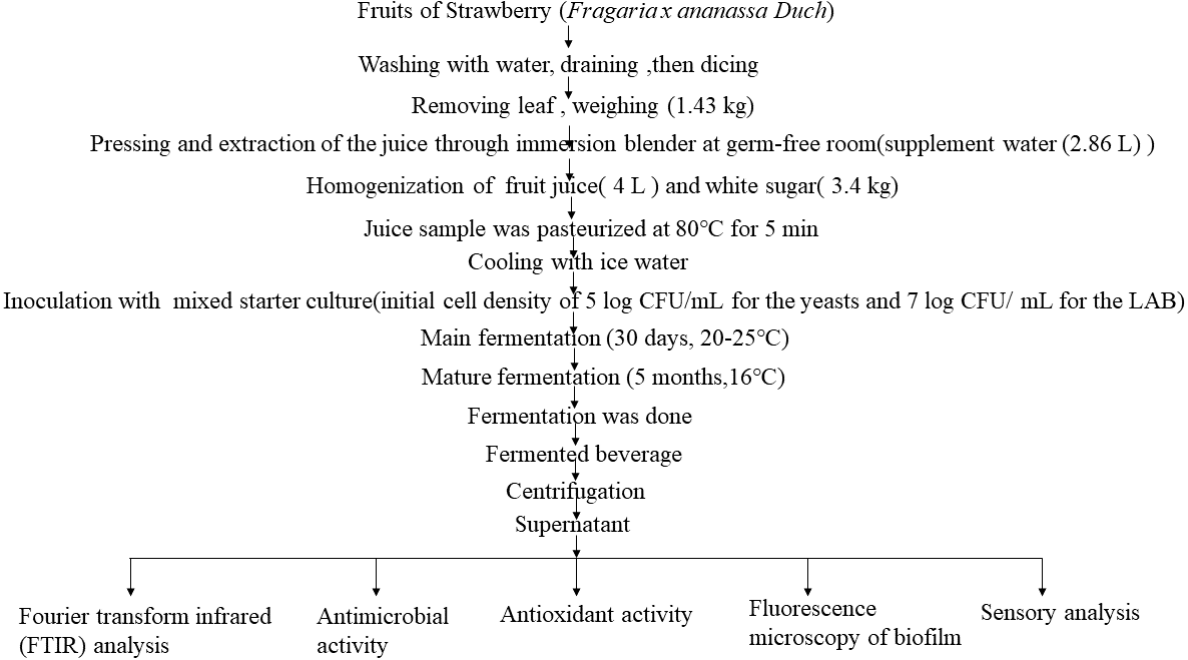
Flow diagram.

The fruit (strawberry) fermentation conducted in this study was performed in the laboratory. Starter cultures (LAB and yeasts) are inoculated into strawberry juice, 30 days for the main fermentation at room temperature (25 °C), 5 months for the maturation is at 16 °C. The whole process is usually slow requiring 6 months. The production process is shown in Fig. 1. Unfermented fresh strawberry juice was used as a control. The fermented strawberry beverage and fresh juice were centrifuged, and the supernatant was used as test sample for research on FTIR, antioxidant capacity (including antioxidant content, free radical scavenging capacity, T-SOD activity, T-AOC), and antibacterial capacity. The fermented beverage-treated biofilms of *Escherichia coli* ATCC 25922 and *Staphylococcus aureus* ATCC 6538 were observed by fluorescence microscopy. Finally, sensory analysis was conducted on the fermentation samples and strawberry juice.

### Raw materials

Fresh strawberries and sugar were procured from a local supermarket in Ya’an City, Sichuan Province, China.

### Starter strains and pathogenic microorganisms

Starter strains of LAB (*Lactobacillus plantarum* and *Lactobacillus bulgaricus)* and yeast (*Saccharomyces cerevisiae*) were obtained from the Sichuan Food Fermentation Industry Research and Design Institute, Chengdu City, Sichuan Province, China. Strains of LAB were incubated in de Man, Rogosa, and Sharpe Medium (MRS), yeast was incubated in extract Extract Peptone Dextrose Medium (YPD) medium.

Four pathogenic microorganisms (*Escherichia coli* ATCC 25922, *Staphylococcus aureus* ATCC 6538, *Pseudomonas aeruginosa* ATCC 9027, and *Bacillus subtilis* ATCC6633) from the College of Life Science, Sichuan Agricultural University, Yaan City, Sichuan Province, China. The bacteria were cultured in Luria-Bertani (LB).

### Preparation of the fermented beverage

Fresh strawberries (1.43 kg) were cleaned, cut and put into the juicer (JYL-C16D/C16V, Joyoung Company Limited), then sterile water (2.86 L) was supplemented to squeeze the strawberries to obtain strawberry juice. Then fruit juice (4 L) was taken, and white sugar (0.64 kg) was added then stirred. Thereafter, the juice sample was pasteurized at 80 °C for 5 min, after pasteurization, the juice sample was immediately cooled with ice water. Subsequently, the starter culture was inoculated into the juice (initial cell density of 5 log CFU/mL for the yeasts and 7 log CFU/ mL for the LAB). Fermentation was conducted in a glass bottle with a total volume of 5 L, of which the substrate occupies 4 L. The main fermentation of the juice was performed for 30 days at 25 °C, and then mature fermentation was conducted for 5 months at 16 °C for a total of 6 months. Finally, the fermented beverage was centrifuged (8000 r/min, for 20 min, 4 °C) by a centrifuge: (Thermo Scientific Sorvall ST 16, Shanghai Fuze Trading Co., Ltd) to obtain supernatants for analysis.

### Fourier transform infrared (FTIR) analysis

The FTIR spectra of the strawberry juice and fermented beverage was examined by an FTIR spectrophotometer (Nicolet IS10, Shanghai Precision Instrument Co., Ltd).

### Antioxidant activity

#### Antioxidant

The total phenol content of the sample was determined by the Folin-Ciocalteu colorimetric method according to previous research, with slight modifications (Abirami, Nagarani & Siddhuraju, 2014). Firstly, 1 mL of Folin-Ciocalteu reagent was taken and then added to the 1 mL of diluted samples. After 3 min, 3.0 mL of the solution of sodium carbonate (20%, w/v) was added. Further, the mixture solution was allowed to incubate for 30 min at 25 °C. Finally, the absorbance of the mixture was measured at 760 nm by a Microplate Reader. The calibration curve was prepared from a standard solution of gallic acid (ranging from0 to 0.035 mg/mL). The total phenol content was expressed by the equivalent of gallic acid, which was calculated according to the calibration curve of gallic acid. The calibration curve equation was *A* = 15.4867C−0.0036 (*R*^2^ = 0.9970).

The total flavonoids content of the sample was determined by the Aluminum chloride colorimetric method according to previous research, with slight modifications (Abirami, Nagarani & Siddhuraju, 2014). Firstly, 0.15 mL of sodium nitrite (5%, m/v) was taken and then added to the 1 mL of diluted samples. After standing for 6 min, 0.3 mL of the solution of aluminum chloride (10%, w/v) was added. After the mixture was allowed to stand for 5 min, 1.00 mL of 1 mol/mL sodium hydroxide was added. Finally, the absorbance of the mixture was measured at 510 nm by a Microplate Reader. The calibration curve was prepared from a standard solution of rutin (ranging from 0 to 0.1mg/mL). The total flavonoids content was expressed by the equivalent of rutin, which was calculated according to the calibration curve of rutin. The calibration curve equation was *A* = 3.0219C+0.0105 (*R*^2^ = 0.9951). T-SOD activity of the sample was determined by the T-SOD assay kit (Jiancheng, Nanjing, China) following the manufacture’s instructions of the kit, respectively.

#### Scavenging free radical capacity

Samples were diluted with distilled water to different concentration (0.08, 0.16, 0. 24, 0. 32, 0.40, 0.48, 0.56, 0.64 mL/mL), use the 0.1 mg/ mL ascorbic acid (Vc) as the control.

DPPH radical-scavenging activity of the sample was determined following the previous study (Yang et al., 2018), with slight modifications. In brief, 0.1 mL of 0.2 mmol/L freshly prepared DPPH solution were added to 0.1 mL of sample and homogenized, then the mixture was incubated at room temperature for 30 min in the dark. The absorbance of the mixture was measured at 517 nm. DPPH radical-scavenging activity is expressed as% and determined as follows the formula:

\begin{eqnarray*}\text{DPPH radical-scavenging activity}~(\text{%})=[1- \frac{{A}_{1}-{A}_{2}}{{A}_{0}} ]\times 100\text{%} \end{eqnarray*}

where *A*_1_ is the absorbance of the sample, *A*_2_ is the absorbance of absolute ethanol instead of DPPH solution, *A*_0_ is the absorbance of solvent instead of the sample solution.

The Hydroxyl radical-scavenging activity of the sample was determined following the previous study, with slight modifications (Xiao et al., 2015). Briefly, 0.2 mL solution of 6 mmol/L FeSO_4_, 0.2 mL solution of 6 mmol/L salicylic acid, and 0.2 mL solution of six mmol/L H_2_O_2_ was taken and added to the 0.2 mL of sample, then make up to 0.5 mL with ethanol. The mixture was reacted at 37 °C for 30 min and its absorbance was measured at 510 nm. Hydroxyl radical-scavenging activity is expressed as% and determined as follows the formula:

\begin{eqnarray*}\text{Hydroxyl radical-scavenging activity}~(\text{%})=[1- \frac{{A}_{1}-{A}_{2}}{{A}_{0}} ]\times 100\text{%} \end{eqnarray*}

where *A*_1_ is the absorbance of the sample, *A*_2_ is the absorbance measured by using ethanol instead of the sample and H_2_0_2_ solution, adding ferrous sulfate and salicylic acid-ethanol solution, *A*_0_ is the absorbance of ethanol instead of sample.

The Superoxide anion radical-scavenging activity of the sample was determined following the previous study (Leong et al., 2008). Firstly, 1 mL of the sample was added to 3 mL of 0.05 mon/L Tris–HCl (pH 8.2) and homogenized, then the mixture was incubated at 25 °C for 30 min. Next, 0.4 mL of 25 mmon/L pyrogallols, which was also pre-warmed to 25 °C, was added to the mixture and incubated for 4 min. Finally, add 0.5 mL of concentrated hydrochloric acid to stop the reaction. The absorbance of the mixture was measured at 325 nm. Superoxide anion radical-scavenging activity is expressed as% and determined as follows the formula:

\begin{eqnarray*}\text{Superoxide anion radical-scavenging activity}~(\text{%})= \frac{{A}_{1}-{A}_{2}}{{A}_{1}} \times 100\text{%} \end{eqnarray*}

where *A*_1_ is the absorbance of the sample, *A*_2_ is the absorbance of distilled water instead of the sample.

ABTS radical-scavenging activity of the sample was determined following the protocol previously described by Tang et al. (2020).

#### T-AOC

The T-AOC of the sample was determined by the Ferric ion reducing antioxidant power method with (T-AOC) assay kit (Jiancheng, Nanjing, China) according to the manufacture’s instructions of the kit.

### Antimicrobial activity

#### Inhibitory zone assay

*In vitro* antimicrobial activity of the sample was determined by the Oxford plate method previously described by Ye, Dai & Hu (2013), with slight modifications. Firstly, 0.2 mL of bacterial suspension of the tested were taken and then was coated on the LB culture plates. Further, three sterile Oxford cups (a stainless cylinder of inner diameter 6.00 mm, outer diameter 7.80 mm, height 10.00 mm) were gently placed on the surface of the culture medium with a tweezer, then 0.2 mL of the sample being sterilized by filtration through 0.22 µm Millipore filter, sterile water (negative control) and 10 mg/mL ampicillin (positive control) were transferred into respectively the Oxford cup and incubated at 37 °C for 12 h. Finally, measurement of the diameter of the inhibition zone (with a micrometer) of the tested pathogenic microorganisms was evaluated for antimicrobial activity.

#### Growth curve

50 µL of *Escherichia coli* ATCC 25922 and *Staphylococcus aureus* ATCC 6538 suspensions with an optical density of 0.6 at 600 nm were added to separate 10 mL sterile LB broth. The treatment group was added 150 µL of fermented beverages filtered through a 0.22 µm Millipore filter, and the control group was added 150 µL of normal saline. The sample was cultured on a shaker at 37 °C and 130 r/min. Sample were taken every 2 h and cultured continuously for 12 h. Then, the OD value was measured at 600 nm with an ultraviolet spectrophotometer. Finally, the growth curves of the treatment group and the normal growth control group were drawn respectively.

#### Determination of MIC and MBC

The test of MIC and MBC of *Escherichia coli* ATCC 25922 and *Staphylococcus aureus* ATCC 6538 was conducted according to the method of Jardak et al. (2017), with slight modifications. The final concentration of the fermented beverage is 0.2, 0.1, 0.05, 0.025, 0.0125, 0.00625, 0.003125, 0.00156, 0.00078, 0.00039 mL/mL.

### Fluorescence microscopy of biofilm

The effect of fermentation on biofilm inhibition was observed by fluorescence microscopy (OLYMPUS BX53, Tianjin Leike Optical Instruments Co., Ltd). This test was conducted according to Jardak et al. (2017).

### Sensory analysis

Sensory analysis of the fermented beverage and strawberry juice was conducted according to Di Cagno et al. (2011). 15 untrained panelists (7 men and 8 women), ranging from 22 to 43 years old from the College of Life Sciences in Sichuan Agricultural University conducted a sensory analysis based on the color, taste, flavor, appearance, and overall impression of the samples. Samples were scored from 1 to 7 (1: dislike extremely; 2: dislike moderately; 3: dislike slightly; 4: neither like nor dislike; 5: like slightly; 6: like moderately; 7: like extremely). The sample is stored in a glass cup at 4 °C with a volume of 15 mL. The sensory evaluation was conducted from 9:00 to noon under white light. The members were given drinking water to rinse their mouths before testing the samples. The sensory evaluation score of the color, taste, flavor, appearance, and overall impression is the average score.

### Data analysis

The results are presented as the mean ± standard deviation of three replicates of each experiment performed. Statistical analysis was carried out using the SPSS 22 software (SAS Institute Inc., USA). Analysis of variance (ANOVA) and Independent-sample *t*-test were used to evaluate the significant difference among various treatments.

**Figure 2:**
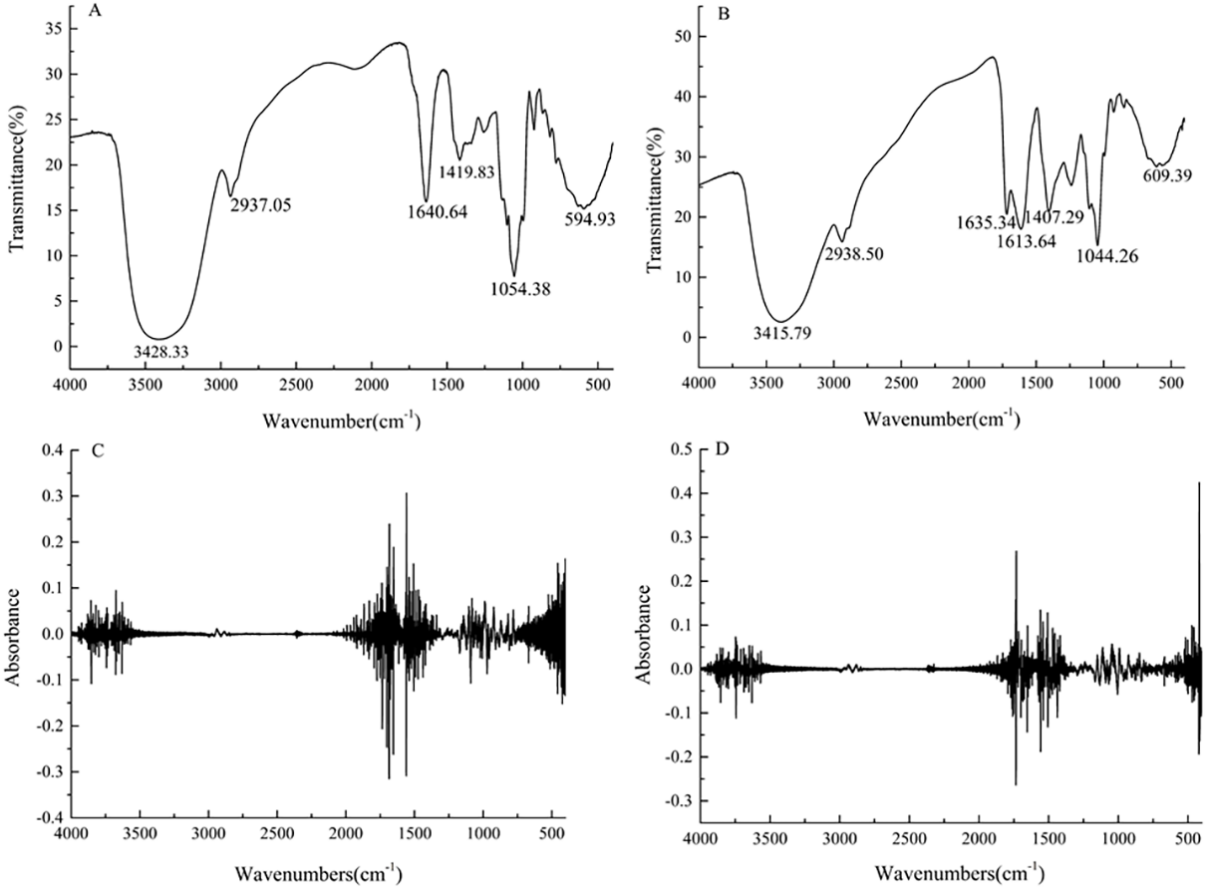
Fourier transform infrared spectra for the strawberry juice (A) and fermented beverage (B) and second-order derivative spectra with strawberry juice (C) and fermented beverage (D).

## Results

### FTIR analysis

FTIR spectrophotometric analysis, which is based on the vibration of functional groups present in the sample when exposed to infrared radiation and was shown in (Figs. 2A–2C). The normalized FTIR spectra were preprocessed second-order derivatives (Figs. 2B–2D). The characteristic absorption peaks at 3,428, 2,937, 1,640, 1,419, 1,054, 594 cm^−1^were observed for strawberry juice. The broad peak at 3428 cm^−1^was attributed to stretching vibration of the -OH functional group. The absorption peaks at 2,937 cm^−1^ were found in the FTIR spectra indicates strong C–H stretching vibration of aliphatic compounds, with methyl (–CH_3_) and methylene(–CH_2_) symmetric and asymmetric stretching vibration. The bands of peaks at 1960–1500 cm^−1^ were assigned to C=C C=N N=N N=O stretching vibration and skeleton vibration of the benzene ring. The absorption peak at 1,419 cm^−1^ can be attributed to the asymmetric stretching vibration of carboxylation (COO-). The absorption peaks at ∼1,040 cm^−1^ represent–C–O alcohols, with a typical ethanol peak.

### Antioxidant activity

#### Antioxidant

The content of the total phenolics, total flavonoids, and the T-SOD activity in the fermented beverage is presented in Table 1. It can be seen from the table that the total phenols and total flavonoids content decreased by 91.1% (*p* < 0.01) and 97.5% (*p* < 0.01), respectively, and T-SOD activity increased by 40.48 U/mL (*p* < 0.01) after six months of fermentation.

#### Free radical scavenging ability

DPPH is a simple and efficient assay is commonly applied to evaluating antioxidant activity. Hydroxyl radical is a very active reactive oxygen species, which could damage the structure of biological macromolecules and cause tissue damage or cell death (Liu et al., 2010). Superoxide anion is produced during normal cellular respiration and plays fundamental roles in cellular physiology with its dysregulation being associated with a variety of diseases (Rahmani, Ghavamipour & Sajedi, 2019). The ABTS radical assay is among the most abundant assays to evaluate antioxidant capacity (Garcia-Leis et al., 2016).

**Figure 3:**
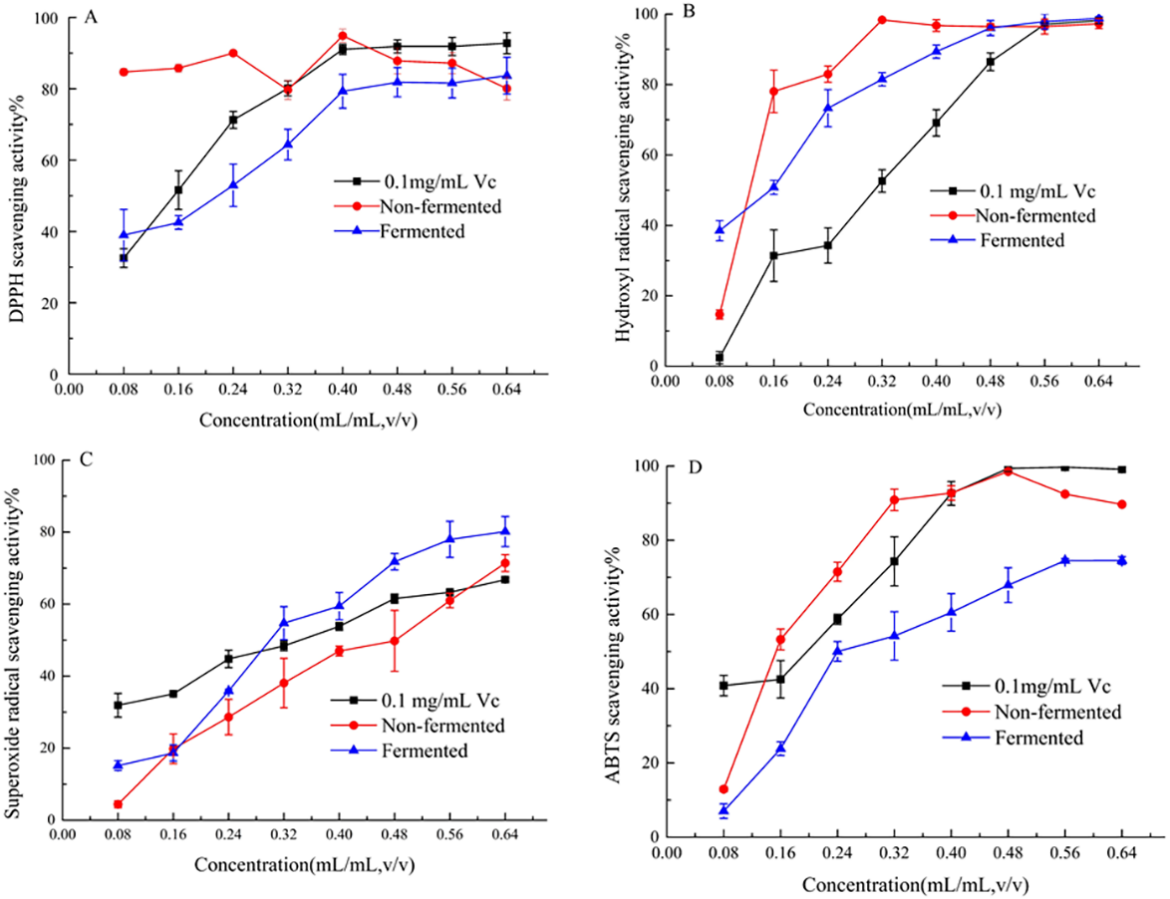
Evaluation of scavenging free radical capacity. DPPH radical-scavenging activity assay (A); Hydroxyl free radical scavenging activity assay (B); Superoxide anion radical-scavenging activity assay (C); ABTS radical-scavenging activity assay (D).

**Figure 4:**
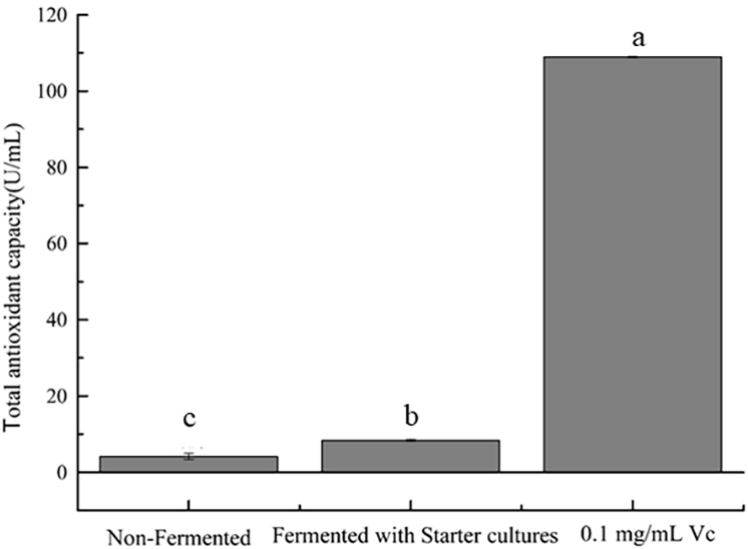
Evaluation of T-AOC. Means that do not share the same letter are significantly different at *p* < 0.05.

In the concentration range of 0.08–0.64 mL/mL, the four free radical scavenging activities of samples are shown in Fig. 3. Samples were found to have the ability to scavenge free radicals and the scavenging rate increased with the increase of the concentration. Figure 3A shows that strawberry juice has the strongest scavenging ability on DPPH free radicals, followed by V_C_, and fermented beverage was the weakest. The maximum value of DPPH scavenging rate was 83.70% and 94.87% of strawberry juice and fermented beverage was found at a concentration of 0.40 mL/mL, respectively. At this concentration, the scavenging rate of V_C_ on DPPH free radicals was 91.05%. Fig. 3B shows that the scavenging ability of strawberry juice and fermented beverage on hydroxyl radicals is stronger than that of V_C,_ which increases from 14.68% to 96.46% and 38.50% to 96.06%, respectively. And V_C_ increases from 31.90% to 66.81%. When the concentration range is 0.48 mL/mL to 0.64 mL/mL, the scavenging rate of hydroxyl radicals of the two samples is almost the same. However, the scavenging rate of fermented beverage on superoxide anions free radical increase from 15.15% to 80.17% is stronger than that of strawberry juice from 4.14% to 71.41%, and also it is stronger than V_C_ in the range from 0.32 mL/mL to 0.64 mL/mL (Fig. 3C). In the ABTS assay, the scavenging rate of fermented beverage on ABTS 6.99% to 74.59% lower than strawberry juice 12.91% to 89.68%, and the scavenging rate of V_C_ has always been higher than that of fermented beverage (Fig. 3D). To further determine the antioxidant capacity of fermented beverage, we also measured its T-AOC, and the results showed that the T-AOC of fermented beverage (8.43 U/mL) was higher than that of strawberry juice (4.15 U/mL), even though they were all lower than the V_C_ (108.90 U/mL) (Fig. 4).

**Table 1:** Analysis of antioxidants in the strawberry fermented beverage.

Parameter	Non-fermented	Fermented
Total phenolic(mg/mL)	3.47 ± 0.02	0.28 ± 0.03^**^
Total flavonoids(mg/mL)	0.40 ± 0.05	0.01 ± 0.001^**^
T-SOD(U/mL)	80.99 ± 1.95	121.47 ± 1.02^**^

**Notes.**

Values are expressed as mean (*n* = 3) ±SD; Independent-sample *t*-test used to evaluate the significant difference among various treatments. Differences were considered to be significant at *p* < 0.01 (**).

Non-fermented corresponds to strawberry juice; Fermented corresponds to fermented beverages. The following occurrences all mean the same thing.

### Antibacterial activity of strawberry fermented beverage

#### Inhibitory zone assay

In this study, *Bacillus subtilis* ATCC6633, *Staphylococcus aureus* ATCC 6538, *Escherichia coli* ATCC 25922 and *Pseudomonas aeruginosa* ATCC 9027 were used as indicator bacteria. The inhibitory zone value of strawberry juice and fermented beverage is shown in Table 2 The represented that the fermented beverage exhibited acceptable antibacterial effects on the four pathogenic microorganisms compared with strawberry juice. Concerning the size of the inhibition zone, the results also showed that the fermented beverage has a more pronounced antibacterial effect against *Escherichia coli* ATCC 25922 (28.30 ± 1.80 mm) and *Staphylococcus aureus* ATCC 6538 (26.60 ± 1.65 mm) compared with *Bacillus subtilis* ATCC 25922 (23.13 ± 1.42 mm) and *Pseudomonas aeruginosa* ATCC 9027 (23.87 ± 2.11 mm), thereby, the first two strains were selected for the following research.

**Table 2:** The diameter of the inhibition zone of the strawberry fermented beverage prepared by lactic acid bacteria and yeast on pathogenic microorganisms.

Microorganisms	Inhibition zone diameter(mm)			
	10 mg/mL ampicillin	Sterile water	Non-fermented	Fermented
*Escherichia coli* ATCC 25922	24.50 ± 1.114^a^	0	0	28.30 ± 1.80^a^
*Staphylococcus aureus* ATCC 6538	24.03 ± 0.61^a^	0	0	26.60 ± 1.65^a^^,^^b^
*Pseudomonas aeruginosa* ATCC 9027	22.53 ± 0.25^b^	0	0	23.13 ± 1.42^c^
*Bacillus subtilis* ATCC6633	9.80 ± 0.36^c^	0	0	23.87 ± 2.11^b^^,^^c^

**Notes.**

Values are expressed as mean (*n* = 3) ±SD; Analysis of variance (ANOVA) was used to evaluate the significant difference among various treatments, with the criterion of *p* < 0.05.

Means that do not share the same letter are significantly different at *p* < 0.05.

#### Growth curve

**Figure 5:**
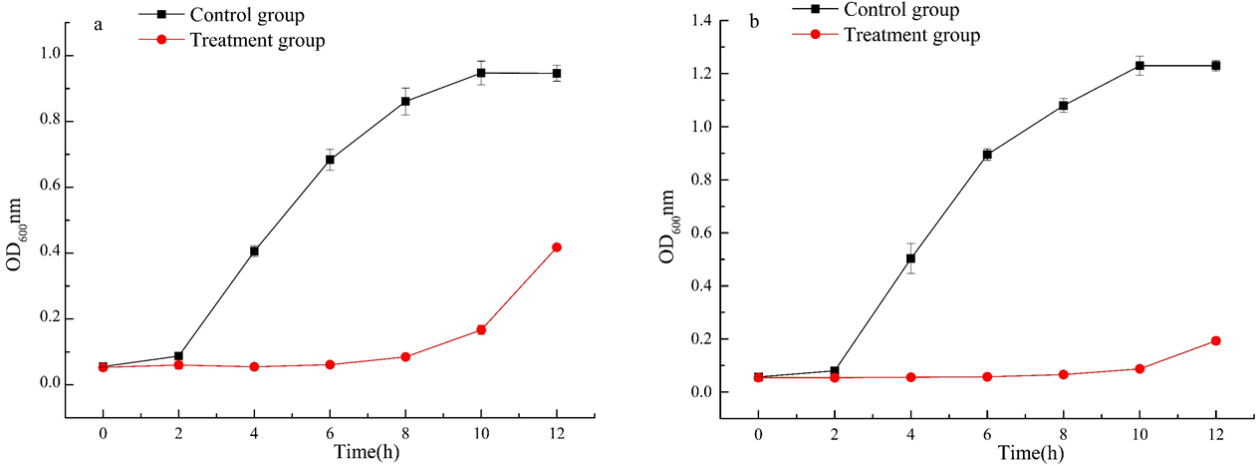
Effects of fermented beverage on growth curve of *Escherichia coli* ATCC 25922 (A) and *Staphylococcus aureus* ATCC 6538 (B).

The bacterial growth curve is an essential representation for characterizing bacteria metabolism within a variety of media compositions. To further demonstrate the antibacterial ability of fermented beverage against *Escherichia coli* ATCC 25922 and *Staphylococcus aureus* ATCC 6538, the bacterial growth curves under the influence of fermented beverage were measured within 12 h. The results showed that the growth rate of the two pathogenic microorganisms, in the medium supplemented with a fermented beverage, was significantly slowed down compared with the control group within 12 h. We speculated fermented beverage has a good antibacterial effect against *Escherichia coli* ATCC 25922 (Fig. 5A) and *Staphylococcus aureus* ATCC 6538 (Fig. 5B), which may be that the presence of fermented beverage in the medium inhibits the growth of microorganisms, resulting in a decrease in the total number of microorganisms and a decrease in the optical density value at OD600.

#### Determination of MIC and MBC

The MIC and MBC assays determined that that the MIC of fermented beverage against *Escherichia coli* ATCC 25922 and the *Staphylococcus aureus* ATCC 6538 were 0.05 mL/mL and 0.025 mL/mL, respectively. whereas the MBC increased, both were 0.2 mL/mL.

### Fluorescence microscopy

**Figure 6:**
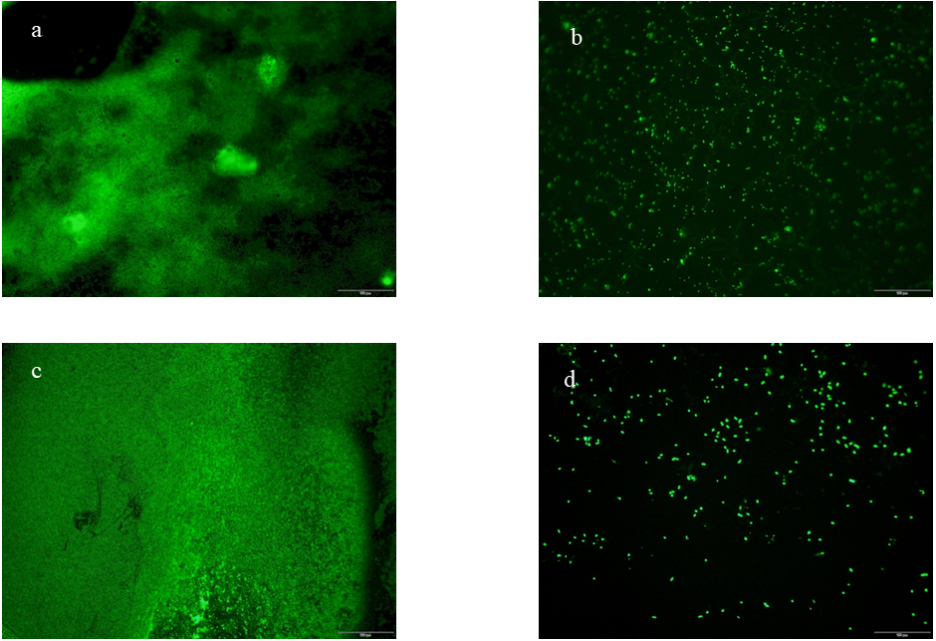
Fluorescence microscopy with biofilm coverslips. Biofilms were stained with acridine orange. Barequals 100 µm. *Escherichia coli* ATCC 25922 biofilm (A), control coverslip; *Escherichia coli* ATCC 25922 biofilm treated with 0.15 mL/mL fermented beverage (B); *Staphylococcus aureus* ATCC 6538 biofilm (C), control coverslip; *Staphylococcus aureus* ATCC 6538 biofilm treated with 0.15 mL/mL fermented beverage (D).

To confirm the ability of fermented beverage to the anti-biofilm formation of *Escherichia coli* ATCC 25922 and *Staphylococcus aureus* ATCC 6538*,* we use a fluorescence microscope to visually demonstrate the inhibitory effect of fermented beverages on biofilm. Figure 6 shows four anti-adhesion images obtained from untreated and fermented beverage-treated biofilm stained with A pyridine orange and observed by a fluorescence microscope. This image shows the difference in biofilm formation between untreated (Figs. 6A and 6C) and treated (Figs. 6B and 6D). It can be seen that the green fluorescent area is greatly reduced after the fermented beverage is treated, and a large number of single colonies appear. It is preliminarily speculated that the fermented beverage has an antiadhesive effect.

### Sensory analysis

In this study, strawberry juice and fermented beverage were sensory evaluated.

**Figure 7:**
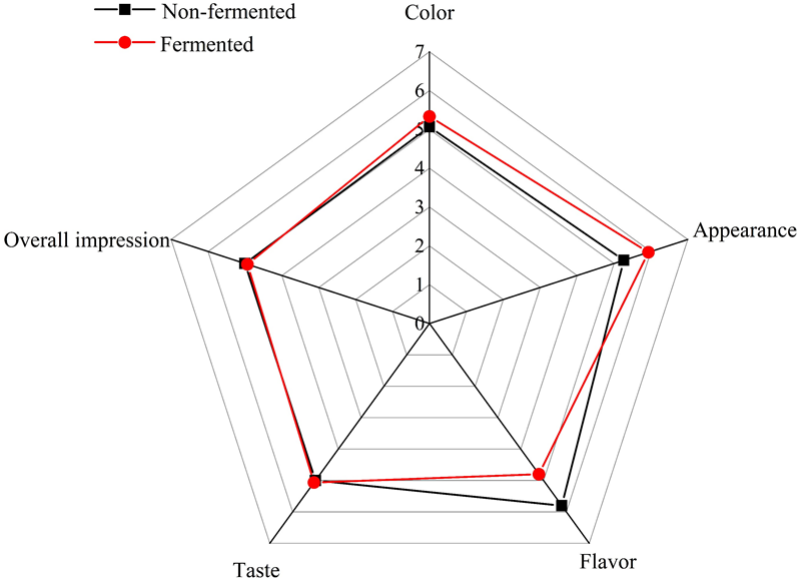
Sensory evaluation of color, appearance, flavor, taste and over impression of strawberry juice and fermented beverage.

Compared to strawberry juice, the average scores of fermented beverage in color, appearance and taste increased by 0.26, 0.66 and 0.07, respectively. The overall impression and flavor decreased by 0.07 and 0.7. Figure 7 shows these results by exhibiting that the flavor and appearance average scores were more variable between the strawberry juice and fermented beverage than the average scores from other attributes, such, color, taste, and overall impression.

## Discussion

Fruit fermented beverages have attracted a lot of attention due to their various potential health benefits. However, research on strawberry fermented beverages is rare and incomplete. Given this, this research comprehensively studied the functional groups, antioxidant capacity, antibacterial capacity, and anti-biofilm formation capacity of the strawberry fermented beverage. In addition, considering the commercial potential of the fermented beverage, we also conducted a sensory evaluation on it.

FTIR technology can be used for the general classification and comparison of food materials including wine due to its fast, accurate, and non-destructive advantage. In our study, a characteristic peak appeared at 2,937 cm^−1^, which means the presence of aromatic ester (Refer to Figs. 2A–2C). Vaithilingam et al. (2016) studies have shown that the presence of aromatic primary amine groups is the result of the fermentation of probiotic microorganisms, which means that there is a bacterial protein in the fermentation product, which could affect the antibacterial properties of the fermented product. Ayed, Ben Abid & Hamdi (2017) stated that the deformation vibration of the C-C bond in the phenolic group absorption peak between 1,500 cm^−1^ and 1,400 cm^−1^; the band between 1,542 cm^−1^ and 965 cm^−1^ is attributed to vibration of the C-O, C-N, and C-N, demonstrating the presence of organic acids, sugars, and ethanol in this area; the peak bands at 1,098 cm^−1^ and 995 cm^−1^ are due to OH deformation and C-O stretching in the phenolic group. The fermented beverage showed several novel peaks, suggesting that the active molecules are transformed by the microorganism during the fermentation process.

Many human chronic diseases such as cancer, aging, obesity, etc. are believed to be related to the oxidative damage of reactive oxygen species (ROS) to cells (Hassan, Ghareb & Azhari, 2017), the presence of antioxidants (such as total phenol, flavonoids, and SOD) to maintain a dynamic balance between the generation and removal of free radicals *in vivo* is important. A standard method for assessing the antioxidant capacity of fermented beverages has not been established (Freire et al., 2017). For that reason the antioxidant activity of fermented beverage was comprehensively conducted to evaluate through changes in antioxidant content, free radical scavenging ability, T-SOD activity and T-AOC. An interesting result that was found in our research was that the content of total phenols and total flavonoids were reduced but the T-SOD was improved after fermentation (Refer to Table 1). In addition, the scavenging ability of fermented beverage to DPPH free radicals, hydroxyl free radicals, and ABTS free radicals was reduced, and the scavenging ability of superoxide anion free radicals was enhanced compared with strawberry juice **(**Refer to Fig. 3). Although the content of antioxidants and the ability to scavenge free radicals have changed, to a greater or lesser degree, the T-AOC has been improved (Refer to Fig. 4). This analysis is consistent with pomegranate juice (Mousavi et al., 2013). The reason why the result probably is that the conversion of phenolic compounds into other new substances with antioxidant activity (Oh et al., 2017), and flavonoid glycosides were converted into corresponding glycosides with strong free radical scavenging ability by specific bacterial glycosyl hydrolases during the fermentation, therefore, the antioxidant properties was increased at the end of fermentation (Marsh et al., 2014; Mousavi et al., 2013). In addition, SOD is an intracellular enzyme, and the increase in its activity may also be related to the lysis of yeast during fermentation (Yang et al., 2018), and it also plays an important role in the increase in antioxidant capacity. Different methods of evaluating antioxidants have been widely used because antioxidant compounds can pass through different mechanisms, each with its specific target in the reaction matrix (Fernández-Moriano et al., 2016). The structure of phenolic compounds has different sites with different free radical scavenging activities (Kwaw et al., 2018). Therefore, different chemical reactivity may lead to different degrees of antioxidant capacity in various chemical tests. In the study of the antibacterial ability of strawberry fermented beverage to pathogenic bacteria, we evaluated the inhibitory band value (Refer to Table 2), MIC, MBC and growth curve (Refer to Figs. 5A and 5B), and the results showed that strawberry fermented beverage showed ideal inhibitory activity. Our results are consistent with Hashemi et al. (2017), who showed that the antibacterial properties of sweet lemon juice through fermentation were improved compared to the control (fresh sweet lemon juice). In another study, fermented dragon fruit juice had a good antibacterial effect on *Escherichia coli, Salmonella Typhimurium*, *Pseudomonas aeruginosa*, and *Staphylococcus aureus* (Muhialdin et al., 2020). Indeed, a large number of previous studies have shown that fermentation process can improve the antibacterial ability of some fruit juices (Hashemi et al., 2017; Vanajakshi et al., 2015; Zubaidah et al., 2018). The antibacterial property of fermented beverages is affected by many factors. The antibacterial potential of phenols mainly lies in the inhibition of bacterial cell wall synthesis, protein synthesis and nucleic acid synthesis (Reygaert, 2014). Flavonoids can destroy the interaction between acyl-homoserine lactone and their receptor, and can also inhibit the surface adhesion by Gram-positive bacteria (Cushnie & Lamb, 2011). However, in this study, the total phenol content and total flavonoid content decreased after fermentation, so the antibacterial activity may be related to organic acids and other metabolites with antibacterial properties produced during the fermentation process. The antibacterial activity of organic acids including un-dissociated acid enters the cell to release protons in the cytoplasm, thereby reducing the pH in the cytoplasm (Zubaidah et al., 2018) and un-dissociated acids lead to the destruction of the substrate transport system by destroying the electrochemical proton gradient or changing the permeability of the cell membrane (Snijders et al., 1985). In addition, metabolites with antibacterial properties such as hydrogen peroxide or diacetyl and bacteriocin peptidoglycan hydrolase are synthesized to play an antimicrobial role during fermentation (Cushnie & Lamb, 2011). Therefore, the antibacterial activity was improved.

Biofilm is a microbial structure that can cause many problems in the medical field, food industry, and other environments. Therefore, the choice of substances inhibiting biofilm formation is very important. However, there is almost no research on the effect of fermented beverages on the formation of biofilms. In this study, it can be seen that the green fluorescent area is greatly reduced after the fermented beverage is treated (Refer to Fig. 6). Our work is of great significance to the effect of fermented fruit and vegetable beverages on the formation of biofilms. Regarding the strategies of inhibiting the formation of biofilms, a study showed that many plants containing active compounds anti-biofilm formation can inhibit bacterial growth and achieve the purpose of preventing bacterial adhesion (Husain et al., 2015). Furthermore, antibacterial peptides, a type of polypeptide or protein with antibacterial activity encoded by genes and synthesized by ribosomes in the metabolic process of LAB, have the effect of inhibiting the formation of biofilms (De la Fuente-Núñez et al., 2013). Therefore, we speculate that the anti-biofilm formation ability of fermented beverages may be related to strawberry as a fermentation carrier and lactic acid bacteria as a starter.

Finally, the newly developed strawberry fermented beverage was submitted to sensorial analysis to its evaluate consumer acceptance in the future. The result showed that improvement of some sensory attributes such as color, appearance and taste in fermented beverage (Refer to Fig. 7) refer to. However, the score of overall impression and flavor were dropped. In consideration development and consumption of the beverage, adding pulp, flavoring and sweetening agents to the beverage to enhance its taste and flavor thus may increase consumer acceptance in the future (Menezes et al., 2018). In addition, Maldonado et al. (2017) reported that the fermentation substrate affects the development of microorganisms and thus the sensory evaluation of fermented products, so the change of the sensory evaluation of strawberry fermented beverage may be related to changes in the composition of strawberry juice before and after fermentation. The large accumulation of lactic acid produced by lactic acid fermentation forms unpleasant flavor, whereas these low acid formation levels are beneficial to food processing and preservation (Galle et al., 2010). Fermentation causes chemical modification of phenolic substances, especially anthocyanins, which results in a lighter color of fermented beverages than strawberry juice, and the color is closely related to appearance (Ayed, Ben Abid & Hamdi, 2017). The results of this study are consistent with this point, and the appearance score is also improved while the color is improved.

## Conclusion

This study analyzed the potential health benefits of strawberry fermented beverages obtained with LAB and yeast as starters for the first time. Fresh strawberry juice was used as a control, although the content of total phenols and total flavonoids in fermented beverages has decreased, the ability to scavenge DPPH free radicals, hydroxyl free radicals and ABTS free radicals has also decreased, but its T-AOC is still improved. In addition, the fermented beverage also has superior antimicrobial activity against four pathogenic microorganisms, and anti-biofilm formation ability for *Escherichia coli* ATCC 25922, *Staphylococcus aureus* ATCC 6538. This study shows the fermentation with LAB and yeast were combined as starter culture is a promising tool for obtaining fermented beverages with health benefits. The results of this study can provide certain theoretical support for the development and promotion of fermented fruit and vegetable beverages in the future. This fruit fermented beverage may bring good news to the lactose intolerant public.

## Supplemental Information

10.7717/peerj.11974/supp-1 Supplemental Information 1 Raw data The “FTIR” shows the wavenumber and fransmittance of the strawberry juice and fermented beverage. This data was used to prepare Fig. 2. “Antioxidations” shows the contents of total phenols and flavonoids, and T-SOD activity. In addition, it shows the scavenging rate of the Vc, strawberry juice and fermented beverage to DPPH radical, ABTS radical, hydroxyl radical and superoxide radical. This data was used to prepare Fig. 3. “Antibacterial” shows the diameter of the inhibition zone of the strawberry juice and fermented beverage on Escherichia coli ATCC 25922, Staphylococcus aureus ATCC 6538, Pseudomonas aeruginosa ATCC 9027, and Bacillus subtilis ATCC6633. This data was used to prepare Table 2. In addition, it also shows absorbance of Escherichia coli ATCC 25922 and Staphylococcus aureus ATCC 6538 under the treatment of fermented beverage within 0-12 h at OD600 nm. This data was used to prepare Fig. 5. “Sensory evaluation” shows the score of each sensory attribute in the sensory evaluation experiment. This data was used to prepare Fig. 7.

10.7717/peerj.11974/supp-2 Supplemental Information 2 The T-AOC of the Vc, strawberry juice and fermented beverage This data was used to prepare Fig. 4.

10.7717/peerj.11974/supp-3 Supplemental Information 3 The content of total sugar and total acid and pH value This was supplied to answer the reviewer’s question and was not shown in this study.

10.7717/peerj.11974/supp-4 Supplemental Information 4 The content of total phenols and flavonoids, and T-SOD activity This was used for data analyses and to prepare Table 1.

## Additional Information and Declarations

### Data Availability

The following information was supplied regarding data availability: The raw data are available in the Supplemental Files.
